# Prediction of neonatal deaths in NICUs: development and validation of machine learning models

**DOI:** 10.1186/s12911-021-01497-8

**Published:** 2021-04-19

**Authors:** Abbas Sheikhtaheri, Mohammad Reza Zarkesh, Raheleh Moradi, Farzaneh Kermani

**Affiliations:** 1 Health Management and Economics Research Center, School of Health Management and Information Sciences, Iran University of Medical Sciences, Tehran, Iran; 2 Maternal, Fetal and Neonatal Research Center, Tehran University of Medical Sciences, Tehran, Iran; 3 Department of Neonatology, Yas Complex Hospital, Tehran University of Medical Sciences, Tehran, Iran; 4 Family Health Institute, Maternal, Fetal and Neonatal Research Center, Tehran University of Medical Sciences, Tehran, Iran; 5 Health Information Technology Department, School of Allied Medical Sciences, Semnan University of Medical Sciences, Semnan, Iran

**Keywords:** Machine learning, Neonate, Death, Prediction

## Abstract

**Background:**

Prediction of neonatal deaths in NICUs is important for benchmarking and evaluating healthcare services in NICUs. Application of machine learning techniques can improve physicians’ ability to predict the neonatal deaths. The aim of this study was to present a neonatal death risk prediction model using machine learning techniques.

**Methods:**

This study was conducted in Tehran, Iran in two phases. Initially, important risk factors in neonatal death were identified and then several machine learning models including Artificial Neural Network (ANN), decision tree (Random Forest (RF), C5.0 and CHART tree), Support Vector Machine (SVM), Bayesian Network and Ensemble models were developed. Finally, we prospectively applied these models to predict neonatal death in a NICU and followed up the neonates to compare the outcomes of these neonates with real outcomes.

**Results:**

17 factors were considered important in neonatal mortality prediction. The highest Area Under the Curve (AUC) was achieved for the SVM and Ensemble models with 0.98. The best precision and specificity were 0.98 and 0.94, respectively for the RF model. The highest accuracy, sensitivity and F-score were achieved for the SVM model with 0.94, 0.95 and 0.96, respectively. The best performance of models in prospective evaluation was for the ANN, C5.0 and CHAID tree models.

**Conclusion:**

Using the developed machine learning models can help physicians predict the neonatal deaths in NICUs.

**Supplementary Information:**

The online version contains supplementary material available at 10.1186/s12911-021-01497-8.

## Background

The neonatal period is the first 28 days of life, which is the stage of developing physiological adaptations for extra-uterine life. This time is a vulnerable period and the high neonatal mortality rate is due to the high level of vulnerability in this period [1]. Neonatal and children death is a major health indicator [2] and mortality prediction is applied for reviewing and benchmarking, looking the results in neonatal intensive care units (NICUs) and evaluating efficacy [3]. About two-thirds of infant deaths and about half of the under-five deaths occur in neonatal period [4]. Predictions show that between 2019 and 2030, approximately 52 million children under the age of 5 will die, approximately half of whom will be neonate [2]. However, the under-5 mortality rate has declined around the world, but the neonatal mortality rate is still an alarming issue [5].

In order to public health policy-making and management of pregnancy, childbirth and neonate periods, including the proper selection of risk factors and development of selective care pathways for high-risk pregnancies, it is important to predict high-risk neonates [6]. Furthermore, early identification of neonates who are at risk for death can help physicians provide early treatment and has a direct impact on their survival and decreasing their morbidity [7].

Machine Learning (ML) is a subset of Artificial Intelligence (AI), which incorporates all methods that permit machines to learn from data [8]. The expectation of ML is to train machines based on the provided data and algorithms. The machines learn how to make autonomous decisions using large sets of data inputs and outputs [9, 10]. In NICUs, decision-making is a complex and important process, and the use of artificial intelligence and machine learning techniques can improve the quality of neonatal care by providing early warnings to healthcare providers [11].

According to some studies, the use of machine learning methods in predicting the neonatal mortality was promising. For example, Mboya et al. [5] showed that the predictive ability of perinatal death in machine learning algorithms was considerably superior over the logistic regression method. However, despite there are many studies in this field, most of them have been done on specific groups of neonates, such as premature or Very Low Birth Weight (VLBW) neonates or in general settings rather than specifically in the NICUs. For instance, in a cohort study in Tanzania (2020), perinatal death prediction using machine learning models were compared to logistic regression. The results showed that there was no significant difference in perinatal death prediction between machine learning and regression models, except for bagging method. In addition, the machine learning algorithms had a superior net benefit and its predictive ability was greatly higher than regression model [5]. In a 2019 study, researchers in Bangladesh developed and evaluated regression models to predict the risk of neonatal death based on known characteristics in the beginning of pregnancy, beginning of delivery and five minutes after delivery. According to results, the predictive ability of the model was moderate at the beginning of pregnancy (AUC = 0.59). At the beginning of delivery, the predictive ability was significantly better (AUC = 0.73) and at 5 min after birth, the predictive ability was good (AUC = 0.85) [6]. Researchers in Ohio (2018) predicted postoperative neonatal deaths using a superlearning method (including 14 algorithms) and showed that performance of the superlearner algorithm was better than any of the other algorithms alone [12].

As for studies related to NICUs, researchers in Iran (2020) used neural network and logistic regression to predict the probability of mortality in preterm neonates after admission to NICU and showed that neural network with 60 neurons in hidden layer had a more acceptable performance [3]. In 2020, researchers in Finland used 9 different classifiers to predict mortality and morbidity among very low birth weight infants on time series data and clinical variables. The results showed that random forest had the best results compared to the other classifiers in predicting death (AUROC = 0.922 and F1-score = 0.493) [13].

Despite of these models to predict the neonatal death risk, the performance of different algorithms on different datasets has been different. Furthermore, the best AUC on neonatal death obtained from these studies was 0.96 [3].

Unlike the previous studies, which were performed on premature or VLBW neonates [3, 13], or in general settings other than NICUs [5, 6, 12], the present study considers all neonates without birth weight limitation in NICU settings. Furthermore, the majority of previous models were not prospectively applied and evaluated [5, 6, 13]; however, in the current study, we prospectively evaluated our models in a NICU to better examine the performance of the models from a clinical perspective.

On the other hand, according to studies, neonatal mortality is more prevalent in the developing countries and may follow a different pattern, so appropriate models need to be developed in these countries based on the internal conditions. Therefore, the purpose of this study was to present a neonatal death risk prediction model using machine learning algorithms and apply these models in an NICU to predict the neonatal death and compare the results with final status of neonates to evaluate the performance of these models.

## Methods

**Fig. 1:**
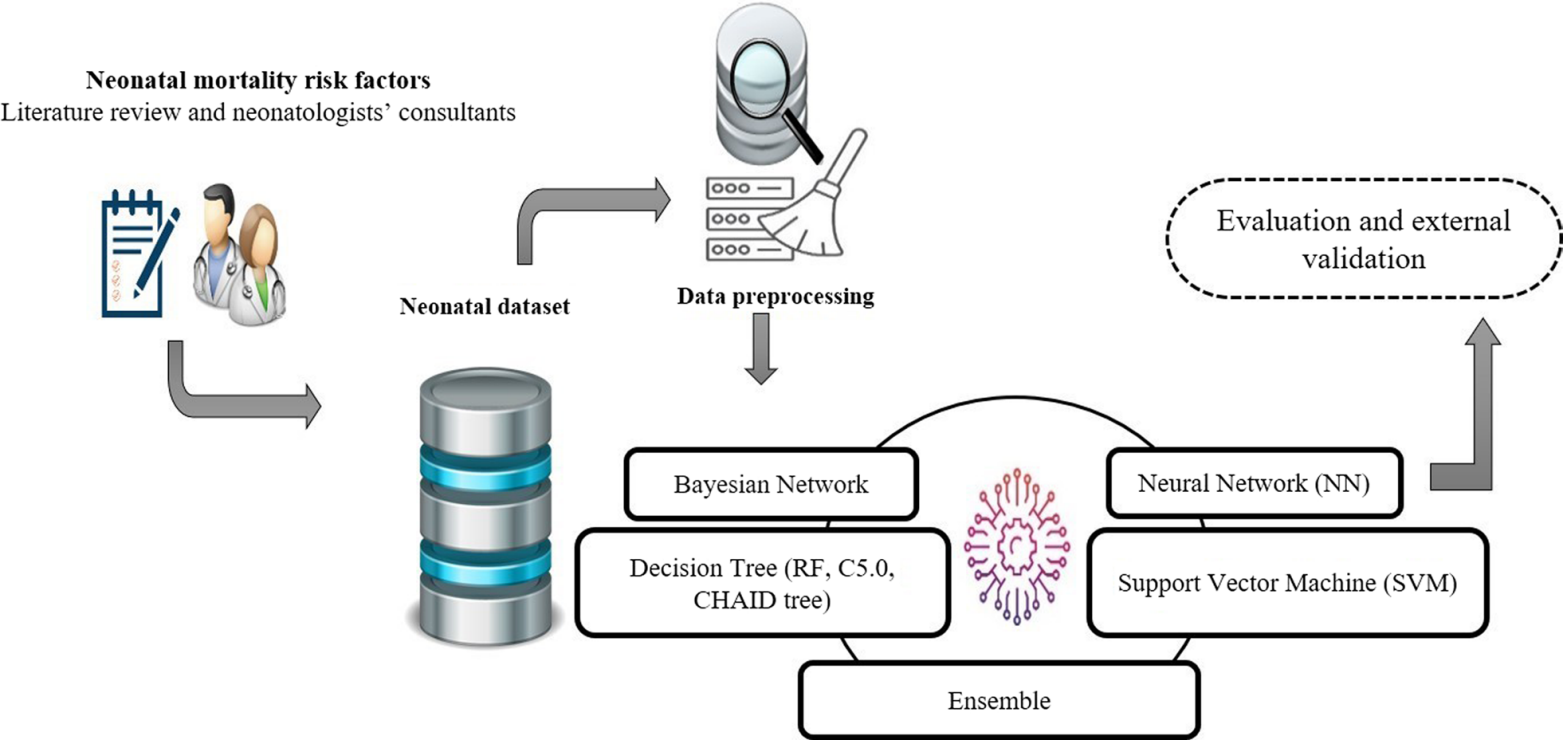
Overall methodology

At first, important risk factors in neonatal death were identified through a literature review, neonatologists’ opinions and different feature selection methods. Then, several machine learning models were developed and evaluated in a prospective study for external validation of models. The overall methodology briefly is described in Fig. 1.

### Identification of the neonatal mortality risk factors

A literature review was conducted to identify neonatal mortality risk factors, which was reported elsewhere [14] and then ultimately 21 important risk factors for neonatal death were identified by neonatologists’ opinions. However, four of these variables were not recorded in the neonatal registry that we used. Therefore, these four variables were excluded and 17 variables were selected for the analysis. There are also different feature selection methods in practice and according to previous studies [15–17], four well-known feature selection methods were applied to identify the most important features from these 17 features. First, we used univariate statistical analysis (non-parametric Mann–Whitney and Chi-square tests) to identify significantly different variables among alive and dead neonates. We considered p-value ≤ 0.05 as our selection criterion and identified 13 features. Some statisticians believe that marginal (p-value < 0.2) significant variables identified from univariate should be included in multivariate analysis. Using this criterion, 14 features were selected. We also used IBM SPSS modeler feature selection node and identified 9 important features. Also, we used ‘CfsSubsetEva’ method in Weka. This method measures the significance of attributes on the basis of predictive ability of attributes and its degree of redundancy. The subsets which are having less inter-correlation but highly correlated to the target class are preferred and according to studies, variables selected by this method have the best results in term of the percentage of correctly classified instances compared to other feature selection methods available in Weka [16, 17] In our dataset, this method identified 6 important features. In Table 1, different feature selection methods are presented.

**Table 1:** Different feature selection results

Feature selection Method	Selected features
Neonatologist opinion	*17 features* BW, GA, Preterm birth, SGA, Parental care, Mother disease, RDS, Steroid therapy, Surfactant administration, Pulmonary hemorrhage, NEC, Congenital malformation, Sepsis, Asphyxia, IVH, Intubation, Ventilation
Non-parametric Mann–Whitney and Chi-square test (p-value < 0.05)	*13 features* BW, GA, Preterm birth, SGA, Mother disease, RDS, Surfactant administration, Pulmonary hemorrhage, Congenital malformation, Sepsis, IVH, Intubation, Ventilation
Non-parametric Mann–Whitney and Chi-square test (p-value > 0.2)	*14 features* BW, GA, Preterm birth, SGA, Mother disease, RDS, Surfactant administration, Pulmonary hemorrhage, Asphyxia, Congenital malformation, Sepsis, IVH, Intubation, Ventilation
IBM SPSS modeler feature selection method	*12 features* BW, GA, Preterm birth, SGA, RDS, Surfactant administration, Pulmonary hemorrhage, Congenital malformation, IVH, Intubation, Ventilation, NEC
CfsSubsetEva method in Weka	*5 features* SGA, Parental care, Pulmonary hemorrhage, Intubation, Ventilation

After implementing several machine learning algorithms on these five sets of features, we found that the better results were obtained for models developed based on 17 and 12 features, however, most models developed based on 17 features had the highest performance. Therefore, we considered this feature set for the further analyses. One of the examples of our experiments on 17 and 12 features on one dataset are presented in Additional file 1: Table 1.

### Neonatal data

The data was collected from a neonatal registry database in “Maternal, Fetal and Neonatal Research Center”, Tehran University of Medical Sciences, Tehran, Iran. This registry contains neonatal records from teaching hospitals in Tehran. We extracted data from 1 May 2017 to 31 July 2018. Based on the previous phase, 17 confirmed neonatal risk factors were extracted from this registry. Our dataset consisted of 1762 records in two classes (dead, n = 138 and survived, n = 1624).

### Data pre-processing

The various models were firstly developed using the original data; however, due to the low sample in “dead” class, the model performance was not acceptable, especially in terms of their specificity. Hence, to obtain the best models, data pre-processing techniques were applied.

#### Missing data imputation

Data imputation is usually used to improve the data quality and performance of machine learning models. For this purpose, many methods are well-documented such as replacing the mean or mode of a class group [18, 19]. In our dataset, 14 of the 17 variables had less than one percent missing values. Details on frequency of missing data in different features in the dead and survived classes are presented in Table 2. We used the mean and the most frequent category of each class (dead vs. survived) to impute continuous and Boolean variables, respectively [20]. The missing values were imputed using IBM SPSS modelers. For example, for “congenital malformation”, most records had a value of "No", so the missing value was replaced with "No".

**Table 2:** Frequency of missing data in different features

Variables	Missing data in dead class (N)	Missing data in survived class (N)	Total
Birth Weight (BW)	1	8	9
Gestational Age (GA)	2	10	12
Prenatal care	0	2	2
Mother disease	0	1	1
Steroid therapy	1	1	2
Surfactant administration	1	0	1
Pulmonary hemorrhage	1	1	2
Congenital malformation	1	1	2
Necrotizing EnteroColitis (NEC)	1	1	2
Sepsis	1	1	2
Intra Ventricular Hemorrhage (IVH)	1	1	2
Asphyxia	1	1	2
Intubation	1	0	1

#### Data balancing

Our dataset was imbalanced regarding to the frequency of each class, and the “dead” class contained only 138 records (7.83%). Hence, we applied two minority oversampling techniques, the Synthetic Minority Over-Sampling Technique (SMOTE) [21] and Adaptive Synthetic (ADASYN) [22] to balance the data using R software version 4.0.4. SMOTE is the most famous method to balance the data to improve random oversampling [23]. This technique works by increasing the sample of minority class. By using this method, majority instances do not change [24] and more data from the minority class is added to the dataset so that the amount of data in the minority and majority classes reaches a more balanced level. Although there are many versions of this technique, most of them do not outperform than the original version, therefore, we relied on the original SMOTE [23]. Furthermore, the goal of ADASYN is to utilize a weighted distribution for different minority class samples relevant to their difficulty level of learning, where more synthetic data is made for minority class samples that are difficult to learn compared to those minority samples that are simple to learn [22].

Based on these two methods, we created new datasets by using R software. We added records to the “dead” class using SMOTE by 3, 4, 5, and 11 times and named them as SMOTE-oversampled dataset1 (live class:1624; dead class:552; ratio: 2.96), SMOTE-oversampled dataset2 (live class:1624; dead class:690; ratio: 2.35), SMOTE-oversampled dataset3 (live class:1624; dead class:828; ratio: 1.96), and SMOTE-oversampled dataset4 (live class: 1624; dead class: 1656, ratio: 1.01). We also used ADASYN (with k = 3) and created the ADASYN-oversampled dataset (live class: 1624; dead class: 1583, ratio: 1.02).

In order to achieve the best results, we implemented different machine learning algorithms on the original data, and the above-mentioned oversampled datasets and compared the performance of the models in terms of confusion matrix measures (AUC and F1 measure).

### Model development

In this phase, we used the selected variables as input variables to develop the machine learning models. The data were randomly splited into two groups: 70% for training and 30% for testing data and then different machine learning algorithms including ANN, decision tree (RF, C5.0 and CHAID tree), SVM and Bayesian network were developed on the original and the oversampled datasets. Also, each algorithm was executed 10 times with different randomly selected train and test sets.

#### Artificial neural network (ANN)

These networks, similar to the natural neural networks, process the input variables through neural processing units [20]. ANN is a set of connected input/output units and each connection has a weight. During the training phase, it adjusts the weights to learn how to predict the output class [25]. There are many kinds of ANNs [26] out of which, we used Radial Basic Function (RBF) and Multiple Layer Perceptron (MLP) networks with different number of processing units in each hidden layer. The selection of the network architecture was done by trial and error, and finally, the network with the best performance was selected.

#### Decision tree

Decision tree classifies data to discrete ones applying tree structure algorithms [25]. The main purpose of these classifiers is to display the structural information stored in the data. This technique generates a decision tree from a set of labeled training samples [18]. The advantages of this method are its ease and speed, ability to handle high dimensional data and its understandable representation [25]. We used the RF, C5.0 and CHAID tree algorithms to construct the decision tree. C5.0 algorithm utilized a pruning method. Also, this method uses a boosting method to build and merge multiple classifiers to deliver improved accuracy [27]. The Chi-squared Detection of Automatic Interaction (CHAID) tree is one of the oldest decision trees for prediction made by repeatedly splitting the subset space into two or more subgroups [28, 29]. CHAID investigates the relationship between a dependent variable and the predictors by maximizing the significance of a Chi-square statistics [30, 31].

RF is one of the most famous machine learning techniques for prediction problems [32]. RF is an Ensemble method that developed multiple decision trees through bootstrap aggregation. Each time an input is supplied to RF, each of the developed decision trees is passed on to that input. Every tree independently predicts a classification and "votes" for the corresponding class. The overall RF forecast is determined by the majority of the votes. Inherently, this combined vote of multiple decision trees is less noisy and less prone to outliers than a single decision tree [13, 33, 34].

#### Support vector machine (SVM)

SVM is an appropriate technique for binary classification. This method is very popular due to its features such as dealing with complex nonlinear data points in the health field. SVM is one of most accurate methods and is less prone to over-fitting than other methods [25, 35]. Furthermore, it is a suitable classifier without the need for any prior knowledge and has high precision and robustness [36, 37]. In addition to linear problems, SVM can also be used as a nonlinear kernel function. The most common kernel functions in SVM include Linear, Polynomial and RBF [25]. In this study, we developed Linear, RBF and Polynomial kernel functions and selected the model with the best performance.

#### Bayesian network

We also applied the Bayesian Network algorithm. These networks are known as statistical classifiers which predict the probability of membership of a given sample in a specific class. Accuracy and speed of this network is high for large databases [38, 39] and the performance of this classifier is also robust [40].

#### Ensemble model

An Ensemble model is one of the methods to increase the classification accuracy. In this technique, a classification model that combines several classification methods is selected. Each classifier returns its vote and the final result is determined by calculating the frequency of votes by each individual classifier [18]. In the current study, the performance measures of different models were reviewed, the best models were selected and combined by two Ensemble methods including Ensemble-Confidence weighted voting and Voting method and their results were compared with each individual models.

### Prospective evaluation and external validation

In order to perform external validation, we conducted a one-month prospective study in the NICU of “Yas” hospital affiliated with Tehran University of Medical Science. We applied our models to predict outcomes of all neonates admitted to this NICU from 20 April 2020 to 20 July 2020 (92 neonates) and then followed them up to their discharge or death (dead, n = 18 and survived, n = 74) and compared the model results with the actual final status of these neonates.

### Implementation and data analysis

We used Statistical Package for the Social Sciences (SPSS) version 23 and Waikato Environment for Knowledge Analysis (Weka) software to analyze the data and identify the important features, respectively. Moreover, we used R software version 4.0.4 and IBM SPSS Modeler version 18 to balance the data and develop the machine learning models, respectively. In this regard, we used confusion matrix and performance measures including accuracy, precision, sensitivity, specificity, F-Score and AUC.

## Results

### The included variables

Based on neonatologist’s opinions, 21 important risk factors were identified and four of them were excluded from the analysis due to high missing data in the dataset. Then, different feature selection methods were applied on identified risk factors (Table 1).

### Description of the neonates

Table 3 indicates the distribution of the quantitative and qualitative variables for all neonates and also dead and survived ones.

**Table 3:** Distribution of qualitative and quantitative features in the original dataset

Variables	Values		Dead (Mean ± SD)	Survived (Mean ± SD)	All neonates (N)
*Quantitative features*					
Birth Weight (BW)	400–6509		1643.5 ± 1083.8	2566** ± **845.5	1762
Gestational Age (GA)	155–281		216 ± 34. 3	249.4 ± 24.1	1762

Variables	Values	Dead N (%)	Survived N (%)	All neonates (N)
*Quantitative features*				
Preterm birth	Yes	111 (80.4)	884 (54.4)	995 (56.5)
	No	27 (19.6)	740 (45.6)	767 (43.5)
Small for Gestational Age (SGA)	Yes	44 (31.9)	318 (19.6)	362 (20.5)
	No	94 (68.1)	1306 (80.4)	1400 (79.5)
Prenatal care	Yes	132 (95.7)	1580 (97.3)	1712 (97.2)
	No	6 (4.3)	44 (2.7)	50 (2.8)
Mother disease	Yes	26 (18.8)	341 (21)	367 (20.8)
	No	112 (81.2)	1283 (79)	1395 (79.2)
Respiratory Distress Syndrome (RDS)	Yes	77 (55.8)	474 (29.2)	551 (31.3)
	No	61 (44.2)	1150 (70.8)	1211 (68.7)
Steroid therapy	Yes	1 (0.7)	8 (0.5)	9 (0.5)
	No	137 (99.3)	1616 (99.5)	1753 (99.5)
Surfactant administration	Yes	90 (65.2)	328 (20.2)	418 (23.7)
	No	48 (34.8)	1296 (79.8)	1344 (76.2)
Pulmonary hemorrhage	Yes	30 (21.7)	4 (0.2)	34 (1.9)
	No	108 (78.3)	1620 (99.8)	1728 (98.1)
Congenital malformation	Yes	62 (44.9)	335 (20.6)	397 (22.5)
	No	76(55.1)	1289 (79.4)	1365 (77.5)
Necrotizing EnteroColitis (NEC)	Yes	6 (4.3)	30 (1.8)	36 (2)
	No	132 (95.7)	1594 (98.2)	1726 (98)
Sepsis	Yes	54 (39.1)	753 (46.4)	807 (45.8)
	No	84 (60.9)	871 (53.6)	955 (54.2)
Intra Ventricular Hemorrhage (IVH)	Yes	41 (29.7)	235 (14.5)	276 (15.7)
	No	97 (70.3)	1389 (85.5)	1486 (84.3)
Asphyxia	Yes	5 (3.6)	29 (1.8)	34 (1.9)
	No	133 (96.4)	1595 (98.2)	1728 (98.1)
Intubation	Yes	78 (56.5)	95 (5.8)	173 (9.8)
	No	60 (43.5)	1529 (94.2)	1589 (90.2)
Ventilation	Yes	104 (75.4)	168 (10.3)	272 (15.4)
	No	34 (24.6)	1456 (89.7)	1490 (84.6)

The mean of BW was 2323.2 gr (1643.5 gr in dead and 2566 gr in survived neonates). Furthermore, the mean of GA was 240.49 days (216 and 249.4 days in dead and survived neonates, respectively). 56.5% of all neonates were preterm and 20.5% were SGA. Additionally, 97.2% of mothers received routine perinatal care during pregnancy (97.3% in survived vs. 95.7% in dead neonates) and only 20.8% of mothers suffered from chronic diseases such as gestational and chronic diabetes, chronic and gestational hypertension and other diseases during pregnancy; 31.3% of neonates had RDS (29.2% in survived vs. 55.8% in dead neonates), and steroid and surfactant administration were seen only in 0.5% and 23.7% of all neonates, respectively. There was also 1.9% pulmonary hemorrhage (0.2% in survived vs. 21.7% in dead neonates), heart disease (22.5%), NEC (2%), sepsis (45.8%) and IVH (15.7%) in neonates. Additionally, the most of neonates had no asphyxia (98.1%), intubation (90.2%) and ventilation need (84.6%).

### The machine learning algorithms and their evaluation

The performance of selected models on the original data indicated that the specificity was not appropriate mainly because of our imbalanced dataset. Therefore, we initially created 5 oversampled datasets. Considering that SMOTE-oversampled dataset1 and SMOTE-oversampled dataset4 had the best results (based on AUC and F-score) for the most models, we only report our results for the original, SMOTE-oversampled dataset1, SMOTE-oversampled dataset4 and ADASYN-oversampled dataset in Table 4. The details of the results for the other oversampled data are presented in Additional file 1: Table 1.

**Table 4:** Performance measures of the selected machine learning models on original, SMOTE-oversampled dataset1, SMOTE-oversampled dataset4 and ADASYN-oversampled dataset

Model	Data	Accuracy	Precision	Specificity	Sensitivity	F-score	AUC
RF	Original data	0.91	0.97	0.62	0.94	0.95	0.90
	SMOTE-oversampled dataset1	0.92	0.98	0.94	0.92	0.95	0.97
	SMOTE-oversampled dataset4	0.94	0.96	0.92	0.97	0.94	0.98
	ADASYN-oversampled dataset	0.96	0.99	0.92	0.99	0.95	0.96
ANN	Original data	0.93	0.95	0.43	0.98	0.97	0.93
	SMOTE-oversampled dataset1	0.91	0.94	0.84	0.94	0.94	0.96
	SMOTE-oversampled dataset4	0.90	0.95	0.85	0.96	0.90	0.96
	ADASYN-oversampled dataset	0.88	0.91	0.86	0.91	0.88	0.95
C5.0	Original data	0.94	0.96	0.47	0.98	0.97	0.82
	SMOTE-oversampled dataset1	0.92	0.96	0.90	0.93	0.95	0.94
	SMOTE-oversampled dataset4	0.94	0.96	0.90	0.97	0.93	0.93
	ADASYN-oversampled dataset	0.95	1	0.90	1	0.94	0.97
SVM	Original data	0.94	0.96	0.55	0.97	0.97	0.90
	SMOTE-oversampled dataset1	0.94	0.97	0.90	0.95	0.96	0.98
	SMOTE-oversampled dataset4	0.95	0.98	0.92	0.96	0.95	0.98
	ADASYN-oversampled dataset	0.94	0.95	0.82	0.93	0.88	0.97
Bayesian network	Original data	0.94	0.97	0.66	0.96	0.96	0.90
	SMOTE-oversampled dataset1	0.90	0.95	0.86	0.91	0.93	0.95
	SMOTE-oversampled dataset4	0.89	0.89	0.87	0.90	0.88	0.96
	ADASYN-oversampled dataset	0.88	0.89	0.86	0.89	0.88	0.94
CHAID tree	Original data	0.94	0.95	0.38	0.98	0.98	0.93
	SMOTE-oversampled dataset1	0.90	0.96	0.88	0.90	0.93	0.96
	SMOTE-oversampled dataset4	0.90	0.96	0.83	0.96	0.89	0.95
	ADASYN-oversampled dataset	0.88	0.89	0.86	0.90	0.88	0.95
Ensemble	Original data	0.94	0.96	0.48	0.98	0.97	0.94
	SMOTE-oversampled dataset1	0.92	0.96	0.88	0.93	0.95	0.98
	SMOTE-oversampled dataset4	0.95	0.97	0.84	0.97	0.95	0.98
	ADASYN-oversampled dataset	0.95	0.98	0.90	0.96	0.94	0.98

According to the Table 4, most models developed on SMOTE-oversampled dataset1 shows slightly better results. Among RF models, RF with 100 built models and 10 maximum tree depths had the best accuracy (0.92) and AUC (0.97). Top RF decision rules are presented in Additional file 1: Table 2. Additional file 1: Table 3 shows different neural network architectures. We found that the best performance in ANNs was obtained in MLP network model with 17 input variables and 9 units in one hidden layer with accuracy (0.91) and AUC (0.96) (Additional file 1: Fig. 1). In addition, the best result for C5.0 was observed in the tree with 11 levels tree depth in terms of the accuracy (0.92) and AUC (0.94), respectively. The C5.0 decision rules are presented in Additional file 1: Table 4. As for SVM, we found that RBF function with stopping criteria = 1.0E-3, Regularization parameter (C) = 10, Regression precision = 0.1 and RBF Gamma = 0.1 had the best accuracy and AUC with values of 0.94 and 0.98, respectively. The details of different SVM models are present in supplement (Additional file 1: Table 5).

**Table 5:** Prospective evaluation results of the selected machine learning models

Model	Accuracy	Precision	Specificity	Sensitivity	F-score	AUC
RF	0.63	0.90	0.72	0.61	0.73	0.81
ANN	**0.86**	0.96	0.83	**0.86**	**0.91**	**0.92**
C5.0	0.84	**0.97**	**0.89**	0.82	0.89	0.91
SVM	0.82	0.94	0.78	0.82	0.88	0.89
Bayesian network	0.67	0.85	0.55	0.71	0.77	0.64
CHAID tree	0.83	**0.97**	**0.89**	0.81	0.88	0.91
Ensemble	0.84	0.95	0.83	0.84	0.89	0.91

The best results for each indicator are bold

**Fig. 2:**
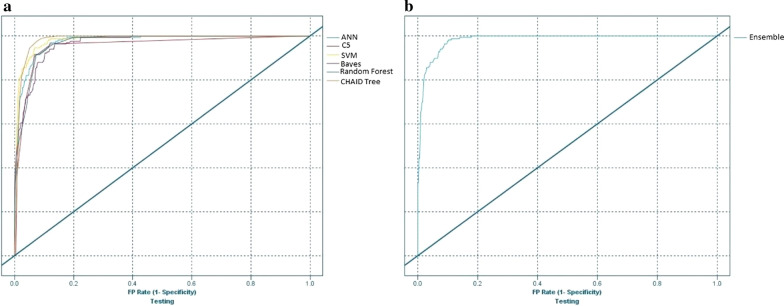
Receiver Operating Characteristic (ROC) curves in **a** selected algorithms **b** Ensemble method on the test data

In addition, for CHAID tree, the 5-depth tree had the best accuracy (0.90) and AUC (0.96). Details on CHAID decision rules are presented in Additional file 1: Table 6. We developed a Bayesian network with two TAN and Markov structures. The network with TAN structure, parameter maximum likelihood learning method, and maximum conditioning size set = 5 had the best accuracy (0.90) and AUC (0.95), respectively on the test data. In Additional file 1 (Fig. 2), the selected Bayesian network structures are shown. Also, all setting, configurations and their values for the best performing models are presented in Additional file 1: Table 7.

**Table 6:** Comparison of our results with related literature

First Author (Reference)	Numbers of variables	List of variables		Study sample	Models	Tool	External validation	Performance evaluation	
Jaskari [13]	10	Heart rate, blood pressure, GA, BW, medical scores SNAP-II and SNAPPE-II, diagnoses of the patients for BPD, NEC, ROP, information on the survival		977 VLBW infants	LR, LDA, QDA, KNN, SVM, 3 different Gaussian process, RF	Matlab	No	AUROC (RF): 0.922 F-Score (RF): 0.477	
Beluzos[42]	23	Mother’s age, BW, 1-min Apgar score, 5-min Apgar score, Robson group classification, number of cesarean deliveries, fetal losses, number of previous gestation, GA, number of live births, number of normal deliveries, birth place code, childbirth care, main worker role, child-birth type (delivery), mother race/skin color, marital status, mother’s years of schooling, week of gestation (by ranges), type of pregnancy, prenatal appointments (by range), newborn presentation type, congenital malformation		698 neonates	XGBoost, LR, RF	Python Programming language (3.6)	Yes	Accuracy (RF): 93% AUC (RF): 0.965	
Rezaeian[3]	15	Maternal age, GA, number of fetus, premature rapture of membrane, maternal preeclampsia, birth type, gender of neonate, BW, birth height, birth head circumference, after birth crying, delivery room breathing, CPR in delivery room, 1th minute APGAR, 5th minute APGAR		1618 premature neonate records	NN, LR	MATLAB R2016a	No	AUC: 95.99% Accuracy: **96.789** Sensitivity: 86.20% Specificity: **98.37%**	
Cooper[12]	68	Not mention		6499 neonates	Superlearning algorithm (14 regression and machine learning algorithms)	SAS version 9.4, R version 3.3.0	Yes	Cross-validated MSE Excellent discrimination (AUC development: 0.91 AUC validation: 0.87) Good calibration in model development/ not good in model validation	
Ravelli[43]	13	GA, fetal gender, use of antenatal corticosteroids, maternal age, parity, caucasian maternal ethnicity, SES, hypertension/pre-eclampsia, PROM, history of preterm birth, bleeding in the second trimester, non-cephalic presentation and level of hospital for delivery (3rd level versus non 3rd level hospital		8500 singleton very preterm infants	Multiple logistic regression, Bootstrapping technique	SAS version 9.2, R version 3.01	No	Discrimination (AUC): 0.83 Accuracy: 65% Calibration: Good calibration	
Our study	17	BW, GA, preterm birth, SGA, prenatal care, mother disease, RDS, steroid therapy, surfactant administration, pulmonary hemorrhage, congenital malformation, NEC, sepsis, IVH, asphyxia, intubation, ventilation		Neonates admitted to NICU	ANN, C5.0, SVM, Bayesian network, Ensemble	IBM SPSS Modeler	Yes	Accuracy (SVM): 94% Precision (RF): **98%** Specificity (RF): 94% Sensitivity (SVM): **95%** F-score (SVM): **0.96** AUC (SVM, ensemble): **0.98**	

The best results in literature are bold

Logistic Regression (LR), Linear Discrimination Analysis (LDA), Quadratic Discrimination Analysis (QDA), K-Nearest Neighborhood (KNN), Random Forest (RF), Area Under ROC(AUROC), Mean Square Error (MSE), Area under Curve (AUC), Classification and Regression Tree (CART), Artificial Neural Network (ANN), Support Vector Machine (SVM), Gestational Age (GA), Birth Weight (BW), Score for Neonatal Acute Physiology-II (SNAP-II), Score for Neonatal Acute Physiology with Perinatal Extension-II (SNAPPE-II), Bronchopulmonary dysplasia (BPD), Necrotizing Enter Colitis ( NEC), Retinopathy of prematurity (ROP), Cardiopulmonary Resuscitation (CPR), Socio-Economic Status (SES), Prelabour Rupture Of the Membranes (PROM), Small for Gestational Age (SGA), Respiratory Distress Syndrome (RDS), Intra Ventricular Hemorrhage (IVH)

Finally, the best RF, ANN, C5.0, SVM, CHAID tree and Bayesian network models were combined using Ensemble methods (Table 4 and Fig. 2). The results indicated that the accuracy and F-score for the SVM was better than the other methods. Comparing different models indicated that the RF had the highest precision and specificity compared to other models. The Ensemble and SVM had the best AUC on the test data. Details on confusion matrix result for each model are presented in Table 8 in Additional file 1.

Among all variables, only the “intubation” was significant in all six models. Then, “GA” was significant in RF, ANN, C5.0, SVM and CHAID tree models. BW, pulmonary hemorrhage and SGA were significant variables that used by at least four models. Congenital malformation, NEC, prenatal care, preterm birth and sepsis were significant variables that used by at least three models. Asphyxia, mother disease and ventilation variables were significant in just two models. Some variables such as steroid therapy, surfactant administration and RDS variables were only significant in one model.

### Prospective evaluation

We conducted a prospective evaluation on the best RF, ANN, C5.0, SVM, CHAID tree, Bayesian network and Ensemble models. Table 5 shows the performance of the models developed based on the SMOTE-oversampled dataset1 in external evaluation. Details on the confusion matrix and the results of the prospective evaluation conducted by other models (developed on other datasets) in Additional file 1: Table 9 and Table 10 . According to the results, most models developed on SMOTE-oversampled dataset1 (except RF) completely outperformed and among them (Table 5), the highest accuracy, sensitivity, F-score and AUC were observed for the ANN; however, C5.0 and CHAID trees had the highest precision and specificity. Therefore, we finally selected models developed based on the SMOTE-oversampled dataset1 on 17 features.

## Discussion

In this study, we developed prediction models for neonatal death in NICU using machine learning algorithms and 17 important variables. Cooper et al. [12] performed the superlearning algorithm on 68 variables. Safdari et al. [41] and Beluzon et al. [42] considered 14 and 23 variables, respectively. Ravelli et al. [43] developed the antenatal prediction of neonatal mortality in very premature infants on 13 variables. Mboya et al. [5] considered 32 predictive variables for perinatal death prediction.

In our study, “intubation” was the only identified important neonatal mortality risk factor were also significant in all six developed predictive models. Studies in Thailand [44], Brazil [45] and Iran [46] also identified this variable as one of the most important risk factors in neonatal death.

GA and BW were also identified as important risk factors by at least four models. Similarly in UK [47], Ethiopia [48],China [49], Brazil [50], Iran [51], Mexico [52], Finland [13] and Brazil [53], these two variables were identified as important risk factors for neonatal mortality. Furthermore, a systematic review indicated the importance of these risk factors for neonatal mortality in NICUs; GA and BW were the most cited risk factors for neonatal death [14]. Some risk factors such as “pulmonary hemorrhage” was found in at least four models of our study and also stated in [45, 54–56], but not mentioned in other machine learning studies [13, 43].

We developed RF, ANN, C5.0, SVM, CHAID tree and Bayesian network as well as Ensemble models and found that the SVM had the highest accuracy, F-score and sensitivity than other models. Also, SVM and Ensemble methods resulted in the highest AUC. The best performance in terms of specificity and precision was for RF. Additionally, the results from prospective evaluation showed the highest accuracy, sensitivity, F-score and AUC was for the ANN model and the highest precision and specificity was for the C5.0 and CHAID tree. This result indicates that ANN, C5.0 and CHAID tree models are more generalizable and applicable for external data.

There are several studies in this respect. Although the studies have been conducted on different data and are not comparable, but as shown in Table 6, Jaskari’s study [13] for predicting the neonatal mortality showed AUC (0.922) and F-score (0.477) for RF classifier. Beluzos et al. [42] proposed a novel support decision method to classify newborns based on their neonatal mortality risk and indicated that the accuracy and AUC were 93% and 0.965, respectively. Rezaeian et al. [3] developed models for prediction of mortality of premature neonates and presented AUC (95.99%), accuracy (96.79%), sensitivity (86.20%) and specificity (98.37%). Cooper’s study [12] for predicting the postoperative neonatal death showed that the AUC for model development and validation were 0.91 and 0.87, respectively. Ravelli et al. [43] developed a model to predict neonatal mortality in very premature infants and indicated that the AUC and accuracy were 0.83 and 0.65, respectively.

Because of different datasets, and targeted neonates, comparing our results with other studies is difficult; however, in general, in comparison with the best results from previous studies, we achieved the highest AUC (98% vs. 95.99% [3]), sensitivity (95% vs. 86.20% [3]) and F-score (96% vs 0.477 [13]) than other similar studies. The highest accuracy (94% for SVM) and specificity (94% for RF) in our study are much better than Vianna’s study (83% and 62%, respectively) [57] and Ribeiro’s study (88.2% and 91.7%, respectively) [53] but less than Rezaeian’s study (96.79% and 98.37% respectively). It should be mentioned that Rezaeian’s models are only applicable for premature neonates [3]. Among studies focused on all neonates (not premature), our models showed better results.

## Study limitations and future studies

One of the limitations of our study was the necessity to balance the original data. Comparison of models developed on the original (imbalanced) with those developed on the oversampled data showed an improved performance especially in terms of specificity. Although, oversampling technique is well-defined in machine learning [21], it produces artificial data that may effect on the results. Therefore, it is suggested that the study should be replicated with a larger dataset that is more balanced. Also, it is recommended to use different minority oversampling techniques other than “SMOTE” and “ADASYN” to balance dataset and compare the results. Additionally, we excluded some of the variables from our analysis because of unavailability of data. It is highly recommended to consider these variables in future studies. Also, given that this dataset was specific to this study, as far as we know, there are no other studies (methods) on this dataset, therefore we were not able to conduct such a comparison. Developing other models on the same dataset is recommended. In addition, no decision support system has been implemented yet. Indeed, future studies should be focused on developing such systems and evaluating the impact of these models and systems on health outcomes.

## Implication

The main audiences of this study are physicians and neonatologists in NICUs. They can consider the models and the different risk factors that are identified as important factors by these models in their decision making in NICUs. Artificial intelligence researchers and developers who are interested in developing predictive models or decision support systems for neonatal mortality can also use the results of this study to select the best models for the prediction of neonatal death.

## Conclusion

We developed several machine learning-based models including RF, ANN, SVM, C5.0, CHAID tree, Bayesian network and Ensemble methods using different feature selection methods to predict neonatal deaths in NICUs. As a result, models developed using feature selected by neonatologists (17 features). The ANN models had the best results in prospective evaluation. Therefore, it is suggested for implementing on similar projects.

## Supplementary Information

**Additional file 1**. Additional information about the setting and performance of machine learning models.

## Data Availability

The data are not available because of the confidentiality policy of the neonatal registry of the Maternal, Fetal and Neonatal Research Center. Data are however available from the authors upon reasonable request and with permission of Maternal, Fetal and Neonatal Research Center. Dr Kermani should be contacted in case one needs to access the data.

## Abbreviations

AI: Artificial intelligence
ANN: Artificial neural network
AUC: Area under curve
BPD: Bronchopulmonary dysplasia
BW: Birth weight
CPR: Cardiopulmonary resuscitation
GA: Gestational age
IVH: Intra ventricular hemorrhage
ML: Machine learning
MLP: Multiple layer perceptron
NEC: Necrotizing enterocolitis
NICU: Neonatal intensive care units
PROM: Prelabour rupture of the membranes
RBF: Radial basic function
RDS: Respiratory distress syndrome
RF: Random forest
ROP: Retinopathy of prematurity
SNAP-II: Score for neonatal scute physiology-II
SNAPPE-II: Score for neonatal acute physiology with perinatal extension-II
SES: Socio-economic status
SGA: Small gestational age
SVM: Support vector machine
VLBW: Very low birth weight
