# Supplementary material for: Prediction of neonatal deaths in NICUs: development and validation of machine learning models
Source: BMC Med Inform Decis Mak. 2021 Apr 19;21:131. doi: 10.1186/s12911-021-01497-8 (PMC8056638; doi:10.1186/s12911-021-01497-8)
Supplement: Supplementary file 1 — Additional file 1. Additional information about the setting and performance of machine learning models. [file 12911_2021_1497_MOESM1_ESM.docx]

**Prediction of neonatal deaths in NICUs: Development and validation of machine learning models**

Abbas Sheikhtaheri, Mohammad Reza Zarkesh, Raheleh Moradi, Farzaneh Kermani

**Content**

Table 1. The best machine learning models in SMOTE-oversampled dataset1 (17 and 12 features), SMOTE-oversampled dataset2 and SMOTE-oversampled dataset3 test datasets

Table 2. Top RF decision rules (SMOTE-oversampled dataset1, 17 features)

Table 3. The different neural network model architectures on test data (SMOTE-oversampled dataset1, 17 features)

Table 4. C5.0 decision rules (SMOTE-oversampled dataset1, 17 features)

Table 5. The performance details in three different kernel functions on balanced data (test data) for the SVM models (SMOTE-oversampled dataset1, 17 features)

Table 6. CHAID tree decision rules (SMOTE-oversampled dataset1, 17 features)

Table 7. Configurations and their values for the best performing models (SMOTE-oversampled dataset1, 17 features)

Table 8. Confusion matrix results in test data (SMOTE-oversampled dataset1, 17 features)

Table 9. Confusion matrix results in prospective evaluation

Table 10. Prospective evaluation results of on SMOTE-oversampled dataset1, SMOTE-oversampled dataset4 and ADASYN-oversampled dataset

Figure 1. The architecture of the selected neural network

Figure 2. The selected Bayesian network architecture

**Table 1.** The best machine learning models in SMOTE-oversampled dataset1 (17 and 12 features), SMOTE-oversampled dataset2 and SMOTE-oversampled dataset3 test datasets

| **Model** | **Data** | **Accuracy** | **Precision** | **Specificity** | **Sensitivity** | **F-score** | **AUC** |
| --- | --- | --- | --- | --- | --- | --- | --- |
| **RF** | SMOTE-oversampled dataset1 (17 features) | 0.92 | 0.98 | 0.94 | 0.92 | 0.95 | 0.97 |
|  | SMOTE-oversampled dataset1 (12 features) | 0.91 | 0.98 | 0.93 | 0.90 | 0.94 | 0.96 |
|  | SMOTE-oversampled dataset2 (17 features) | 0.93 | 0.98 | 0.95 | 0.92 | 0.95 | 0.97 |
|  | SMOTE-oversampled dataset3 (17 features) | 0.92 | 0.97 | 0.94 | 0.91 | 0.94 | 0.98 |
| **ANN** | SMOTE-oversampled dataset1 (17 features) | 0.91 | 0.94 | 0.84 | 0.94 | 0.94 | 0.96 |
|  | SMOTE-oversampled dataset1 (12 features) | 0.91 | 0.95 | 0.84 | 0.93 | 0.94 | 0.96 |
|  | SMOTE-oversampled dataset2  (17 features) | 0.91 | 0.95 | 0.87 | 0.93 | 0.94 | 0.96 |
|  | SMOTE-oversampled dataset3  (17 features) | 0.91 | 0.93 | 0.867 | 0.93 | 0.934 | 0.96 |
| **C5.0** | SMOTE-oversampled dataset1 (17 features) | 0.92 | 0.96 | 0.90 | 0.93 | 0.95 | 0.94 |
|  | SMOTE-oversampled dataset1 (12 features) | 0.90 | 0.94 | 0.81 | 0.94 | 0.94 | 0.93 |
|  | SMOTE-oversampled dataset2  (17 features) | 0.93 | 0.96 | 0.91 | 0.93 | 0.95 | 0.92 |
|  | SMOTE-oversampled dataset3 (17 features) | 0.94 | 0.96 | 0.92 | 0.95 | 0.96 | 0.95 |
| **SVM** | SMOTE-oversampled dataset1 (17 features) | 0.94 | 0.97 | 0.90 | 0.95 | 0.96 | 0.98 |
|  | SMOTE-oversampled dataset1 (12 features) | 0.92 | 0.95 | 0.84 | 0.95 | 0.95 | 0.96 |
|  | SMOTE-oversampled dataset2 (17 features) | 0.94 | 0.96 | 0.90 | 0.95 | 0.95 | 0.98 |
|  | SMOTE-oversampled dataset3 (17 features) | 0.94 | 0.95 | 0.90 | 0.95 | 0.95 | 0.98 |
| **Bayes** | SMOTE-oversampled dataset1 (17 features) | 0.90 | 0.95 | 0.86 | 0.91 | 0.93 | 0.95 |
|  | SMOTE-oversampled dataset1 (12 features) | 0.89 | 0.94 | 0.83 | 0.91 | 0.93 | 0.95 |
|  | SMOTE-oversampled dataset2 (17 features) | 0.90 | 0.94 | 0.86 | 0.92 | 0.93 | 0.95 |
|  | SMOTE-oversampled dataset3 (17 features) | 0.88 | 0.92 | 0.84 | 0.90 | 0.91 | 0.95 |
| **CHART tree** | SMOTE-oversampled dataset1 (17 features) | 0.90 | 0.96 | 0.88 | 0.90 | 0.93 | 0.96 |
|  | SMOTE-oversampled dataset1 (12 features) | 0.90 | 0.96 | 0.88 | 0.90 | 0.93 | 0.96 |
|  | SMOTE-oversampled dataset2 (17 features) | 0.91 | 0.95 | 0.89 | 0.93 | 0.94 | 0.97 |
|  | SMOTE-oversampled dataset3 (17 features) | 0.88 | 0.89 | 0.80 | 0.92 | 0.91 | 0.96 |
| **Ensemble** | SMOTE-oversampled dataset1 (17 features) | 0.92 | 0.96 | 0.88 | 0.93 | 0.95 | 0.98 |
|  | SMOTE-oversampled dataset1 (12 features) | 0.89 | 0.92 | 0.85 | 0.93 | 0.93 | 0.95 |
|  | SMOTE-oversampled dataset2 (17 features) | 0.92 | 0.95 | 0.88 | 0.93 | 0.94 | 0.98 |
|  | SMOTE-oversampled dataset3 (17 features) | 0.90 | 0.94 | 0.90 | 0.90 | 0.92 | 0.97 |

**Table 2.** Top RF Decision rules (SMOTE-oversampled dataset1, 17 features)

| **Decision Rule** | **Rule Accuracy** | **Forest Accuracy** |
| --- | --- | --- |
|  |  |  |
| (BW > 2970.0) and (Surfactant = No) and (Congenital = No) and (intubation = No) and (BW > 1280.0) => Survived | 1.000 | 1.000 |
| (sepsis = No) and (mechanical_ventilation = No) and (Congenital = Yes) and (intubation = No) and (BW > 1280.0) => Survived | 1.000 | 1.000 |
| (RDS = No) and (intubation = No) and (BW > 3200.0) and (BW > 1280.0) => Survived | 1.000 | 1.000 |
| (BW > 1730.0) and (Surfactant = Yes) and (BW > 915.0) and (intubation = No) and (mechanical_ventilation = No) => Dead | 1.000 | 1.000 |
| (BW > 2650.0) and (Surfactant = No) and (BW > 915.0) and (intubation = No) and (mechanical_ventilation = No) => Dead | 0.989 | 0.989 |

**Table 3**. The different neural network model architectures on test data (SMOTE-oversampled dataset1, 17 features)

| The number of hidden layers | The number of neurons in hidden layers * | Performance measure on test data | | | | | |
| --- | --- | --- | --- | --- | --- | --- | --- |
|  |  | **Accuracy** | **Precision** | **Specificity** | **Sensitivity** | **F-Score** | **AUC** |
| 1 | 7 | 0.91 | 0.94 | 0.8 | 0.94 | 0.949 | 0.949 |
| 1 | 9 | 0.91 | 0.94 | 0.81 | 0.94 | 0.938 | 0.946 |
| 1 | 10 | 0.91 | 0.93 | 0.8 | 0.95 | 0.941 | 0.946 |
| 1 | 11 | 0.91 | 0.93 | 0.79 | 0.95 | 0.939 | 0.955 |
| 1 | 12 | 0.89 | 0.91 | 0.73 | 0.95 | 0.93 | 0.932 |
| 2 | 8-8 | 0.9 | 0.91 | 0.73 | 0.95 | 0.93 | 0.954 |
| 2 | 9-9 | 0.9 | 0.93 | 0.79 | 0.94 | 0.93 | 0.937 |

* The first number is the number of neurons in the first hidden layer and the second number is the number of neurons in the second hidden layer

Area Under Curve (AUC)

**Table 4**. C5.0 decision rules (SMOTE-oversampled dataset1, 17 features)

| **C5 decision rules** |
| --- |
| mechanical_ventilation = Yes  pulmonary_haemorrhage = Yes => Dead  pulmonary_haemorrhage = No  intubation = Yes  NEC = Yes  SGA = Yes => Dead  SGA = No => Survived  NEC = No  SGA = Yes => Dead  SGA = No  Congenital = Yes  IVH = Yes  sepsis = Yes => Survived  sepsis = No => Dead  IVH = No => Dead  Congenital = No => Dead  intubation = No  PretermBirth = Yes  Prenatal_care = Yes  GA <= 209.370 => Dead  GA > 209.370  Chronic_disease = Yes  RDS = Yes  SGA = Yes => Dead  SGA = No => Survived  RDS = No => Dead  Chronic_disease = No => Survived  Prenatal_care = No => Dead  PretermBirth = No => Survived  mechanical_ventilation = No  intubation = Yes  Congenital = Yes => Dead  Congenital = No  GA <= 189.064 => Dead  GA > 189.064 => Survived  intubation = No  GA <= 189  GA <= 161 => Survived  GA > 161  sepsis = Yes => Survived  sepsis = No => Dead  GA > 189  BW <= 1,643  PretermBirth = Yes  RDS = Yes => Survived  RDS = No  Surfactant = Yes  GA <= 204.184 => Survived  GA > 204.184 => Dead  Surfactant = No => Survived  PretermBirth = No => Dead Survived  BW > 1,643 => Survived |

**Table 5.** The performance details in three different kernel functions on balanced data (test data) for the SVM models (SMOTE-oversampled dataset1, 17 features)

|  | SVM-RBF | SVM-Linear | SVM-Polynomial |
| --- | --- | --- | --- |
| **Accuracy** | 0.93 | 0.9 | 0.94 |
| **Precision** | 0.95 | 0.92 | 0.97 |
| **Specificity** | 0.85 | 0.75 | 0.9 |
| **Sensitivity** | 0.96 | 0.95 | 0.95 |
| **F-score** | 0.954 | 0.93 | 0.95 |
| **AUC** | 0.965 | 0.937 | 0.952 |

Support Vector Machine (SVM), Radial Basic Function (RBF), Area Under Curve (AUC)

**Table 6**. CHAID tree decision rules (SMOTE-oversampled dataset1, 17 features)

| **CHAID tree decision rules** |
| --- |
| mechanical_ventilation = No  intubation = No  BW <= 914.737 => Dead  BW > 914.737 and BW <= 1,720  sepsis = No  Surfactant = No => Survived  Surfactant = Yes => Survived  sepsis = Yes => Survived  BW > 1,720  SGA = No  Congenital = No => Survived  Congenital = Yes => Survived  SGA = Yes  Chronic_disease = No => Survived  Chronic_disease = Yes => Survived  intubation = Yes  Congenital = No  GA <= 191.531 => Dead  GA > 191.531 => Survived  Congenital = Yes => Dead  mechanical_ventilation = Yes  intubation = No  pulmonary_haemorrhage = No  PretermBirth = No => Survived  PretermBirth = Yes  Chronic_disease = No => Survived  Chronic_disease = Yes => Dead  pulmonary_haemorrhage = Yes => Dead  intubation = Yes => Dead |

**Table 7**. Configurations and their values for the best performing models (SMOTE-oversampled dataset1, 17 features)

| **SVM**  Stopping criteria: 1.0E-3  Regularization parameter (C): 10  Regression precision (epsilon): 0.1  Kernel type: RBF |
| --- |
| **NN**  Neural network model: Multilayer Perceptron (MLP)  Hidden layer 1: 9  Hidden layer 2: 0  Default combining rule for categorical targets: Voting  Default combining rule for categorical targets: Mean  Number of component model for boosting and or bagging: 10 |
| **Bayesian**  Structure type: TAN  Parameter learning method: maximum likelihood |
| **RF**  Number of models to build: 100  Sample size: 1.0  Maximum number of nodes: 10000  Maximum tree depth: 10  Maximum child node size: 5  Number of bins: 10 |
| **C5.0**  Build model for each split  Output type: decision tree  Cross validate/ number of folds: 10 |
| **CHAID tree**  Maximum tree depth: 5  Stopping rules:  Minimum records in parent branch (%): 2.0  Minimum records in child branch (%): 1.0  Default combining rule for categorical targets: Voting  Default combining rule for continuous targets: Mean  Number of component models for boosting or bagging: 10  Chi-square for categorical target: pearson |
| **Ensemble**  Ensemble method: confidence weighted voting  If voted is tied, select value using: random selection |

**Table 8**. Confusion matrix results in test data (SMOTE-oversampled dataset1, 17 features)

| **TN** | **FP** | **FN** | **TP** | **Model** | |
| --- | --- | --- | --- | --- | --- |
| 163 | 6 | 31 | 406 | Round 1 | **RF** |
| 151 | 5 | 47 | 432 | Round 2 |  |
| 147 | 7 | 48 | 445 | Round 3 |  |
| 163 | 13 | 40 | 444 | Round 4 |  |
| 148 | 7 | 44 | 428 | Round 5 |  |
| 155 | 9 | 36 | 422 | Round 6 |  |
| 148 | 14 | 35 | 457 | Round 7 |  |
| 143 | 14 | 38 | 454 | Round 8 |  |
| 142 | 8 | 41 | 441 | Round 9 |  |
| 164 | 6 | 43 | 453 | Round 10 |  |
| 156 | 13 | 23 | 414 | Round 1 | **SVM** |
| 146 | 10 | 25 | 454 | Round 2 |  |
| 144 | 10 | 22 | 471 | Round 3 |  |
| 158 | 18 | 27 | 457 | Round 4 |  |
| 138 | 17 | 21 | 451 | Round 5 |  |
| 145 | 19 | 20 | 438 | Round 6 |  |
| 134 | 28 | 19 | 473 | Round 7 |  |
| 136 | 21 | 24 | 468 | Round 8 |  |
| 140 | 10 | 26 | 456 | Round 9 |  |
| 156 | 14 | 23 | 473 | Round 10 |  |
| 134 | 35 | 25 | 412 | Round 1 | **NN** |
| 146 | 20 | 32 | 447 | Round 2 |  |
| 136 | 18 | 32 | 461 | Round 3 |  |
| 153 | 23 | 29 | 455 | Round 4 |  |
| 128 | 27 | 32 | 440 | Round 5 |  |
| 138 | 26 | 36 | 422 | Round 6 |  |
| 133 | 29 | 23 | 469 | Round 7 |  |
| 128 | 29 | 36 | 456 | Round 8 |  |
| 119 | 31 | 27 | 455 | Round 9 |  |
| 149 | 21 | 31 | 465 | Round 10 |  |
| 154 | 15 | 30 | 407 | Round 1 | **C5.0** |
| 142 | 14 | 27 | 452 | Round 2 |  |
| 147 | 7 | 42 | 451 | Round 3 |  |
| 155 | 21 | 24 | 460 | Round 4 |  |
| 138 | 16 | 38 | 434 | Round 5 |  |
| 144 | 20 | 35 | 423 | Round 6 |  |
| 149 | 13 | 31 | 461 | Round 7 |  |
| 137 | 20 | 34 | 458 | Round 8 |  |
| 137 | 13 | 27 | 455 | Round 9 |  |
| 146 | 24 | 32 | 464 | Round 10 |  |
| 147 | 22 | 35 | 402 | Round 1 | **Bayes** |
| 138 | 18 | 47 | 432 | Round 2 |  |
| 136 | 18 | 43 | 450 | Round 3 |  |
| 147 | 29 | 41 | 442 | Round 4 |  |
| 128 | 27 | 49 | 423 | Round 5 |  |
| 132 | 32 | 41 | 417 | Round 6 |  |
| 139 | 23 | 31 | 457 | Round 7 |  |
| 133 | 23 | 38 | 454 | Round 8 |  |
| 134 | 15 | 41 | 441 | Round 9 |  |
| 151 | 19 | 42 | 452 | Round 10 |  |
| 139 | 28 | 57 | 430 | Round 1 | **CHAID tree** |
| 143 | 21 | 50 | 462 | Round 2 |  |
| 142 | 12 | 48 | 445 | Round 3 |  |
| 160 | 16 | 46 | 438 | Round 4 |  |
| 135 | 20 | 49 | 423 | Round 5 |  |
| 142 | 22 | 41 | 417 | Round 6 |  |
| 145 | 17 | 32 | 460 | Round 7 |  |
| 137 | 20 | 49 | 443 | Round 8 |  |
| 130 | 20 | 46 | 436 | Round 9 |  |
| 153 | 17 | 51 | 445 | Round 10 |  |
| 152 | 15 | 35 | 470 | Round 1 | **Ensemble** |
| 143 | 16 | 36 | 453 | Round 2 |  |
| 148 | 9 | 29 | 465 | Round 3 |  |
| 144 | 15 | 33 | 443 | Round 4 |  |
| 138 | 17 | 34 | 448 | Round 5 |  |
| 136 | 17 | 33 | 491 | Round 6 |  |
| 144 | 20 | 25 | 445 | Round 7 |  |
| 143 | 18 | 34 | 476 | Round 8 |  |
| 140 | 21 | 42 | 474 | Round 9 |  |
| 25. | 24 | 35 | 456 | Round 10 |  |

**Table 9**. Confusion matrix results in prospective evaluation

| **TN** | **FP** | **FN** | **TP** | **Model** |
| --- | --- | --- | --- | --- |
| 13 | 5 | 29 | 45 | **RF** |
| 14 | 4 | 13 | 61 | **SVM** |
| 15 | 3 | 10 | 64 | **NN** |
| 16 | 2 | 13 | 61 | **C5.0** |
| 11 | 9 | 21 | 51 | **Bayes** |
| 16 | 2 | 14 | 60 | **CHAID tree** |
| 15 | 3 | 12 | 62 | **Ensemble** |

**Table 10**. Prospective evaluation results of models developed on SMOTE-oversampled dataset1, SMOTE-oversampled dataset4 and ADASYN-oversampled dataset

| Model | Data | Accuracy | Precision | Specificity | Sensitivity | F-score | AUC |
| --- | --- | --- | --- | --- | --- | --- | --- |
| RF | SMOTE-oversampled dataset1 | 0.63 | 0.90 | 0.72 | 0.61 | 0.73 | 0.81 |
|  | SMOTE-oversampled dataset4 | 0.59 | 0.97 | 0.94 | 0.50 | 0.66 | 0.87 |
|  | ADASYN-oversampled dataset | 0.84 | 0.98 | 0.81 | 0.94 | 0.89 | 0.89 |
| ANN | SMOTE-oversampled dataset1 | 0.86 | 0.96 | 0.83 | 0.86 | 0.91 | 0.92 |
|  | SMOTE-oversampled dataset4 | 0.63 | 1.0 | 0.54 | 1.0 | 0.70 | 0.85 |
|  | ADASYN-oversampled dataset | 0.70 | 0.98 | 0.64 | 0.94 | 0.77 | 0.90 |
| C5.0 | SMOTE-oversampled dataset1 | 0.84 | 0.97 | 0.89 | 0.82 | 0.89 | 0.91 |
|  | SMOTE-oversampled dataset4 | 0.47 | 0.80 | 0.56 | 0.45 | 0.57 | 0.53 |
|  | ADASYN-oversampled dataset | 0.75 | 0.98 | 0.70 | 0.94 | 0.82 | 0.82 |
| SVM | SMOTE-oversampled dataset1 | 0.82 | 0.94 | 0.78 | 0.82 | 0.88 | 0.89 |
|  | SMOTE-oversampled dataset4 | 0.72 | 0.93 | 0.78 | 0.70 | 0.80 | 0.85 |
|  | ADASYN-oversampled dataset | 0.76 | 0.93 | 0.76 | 0.78 | 0.84 | 0.87 |
| Bayes | SMOTE-oversampled dataset1 | 0.67 | 0.85 | 0.55 | 0.71 | 0.77 | 0.64 |
|  | SMOTE-oversampled dataset4 | 0.49 | 0.87 | 0.84 | 0.39 | 0.55 | 0.69 |
|  | ADASYN-oversampled dataset | 0.65 | 0.88 | 0.65 | 0.63 | 0.75 | 0.64 |
| CHAID | SMOTE-oversampled dataset1 | 0.83 | 0.97 | 0.89 | 0.81 | 0.88 | 0.91 |
|  | SMOTE-oversampled dataset4 | 0.47 | 0.94 | 0.76 | 0.34 | 0.51 | 0.86 |
|  | ADASYN-oversampled dataset | 0.74 | 1.0 | 0.68 | 1.0 | 0.81 | 0.84 |
| Ensemble | SMOTE-oversampled dataset1 | 0.84 | 0.95 | 0.83 | 0.84 | 0.89 | 0.91 |
|  | SMOTE-oversampled dataset4 | 0.64 | 1.0 | 1.0 | 0.55 | 0.71 | 0.83 |
|  | ADASYN-oversampled dataset | 0.80 | 0.97 | 0.78 | 0.89 | 0.87 | 0.88 |


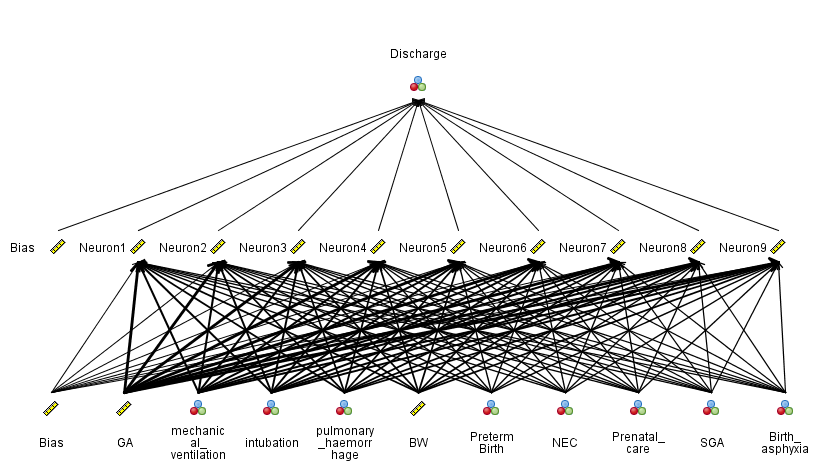


**Figure 1.** The architecture of the selected neural network


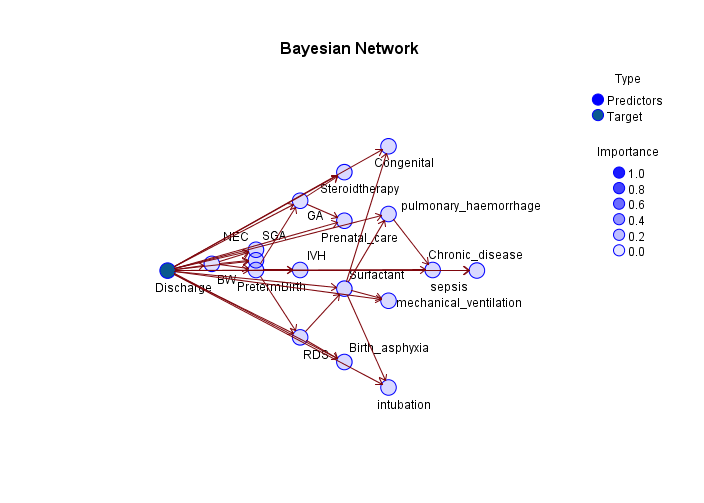


**Figure 2.** The selected Bayesian network architecture
